# Hybrid Silicon Nitride Photonic Integrated Circuits Covered by Single-Walled Carbon Nanotube Films

**DOI:** 10.3390/nano13162307

**Published:** 2023-08-11

**Authors:** Sophia Komrakova, Pavel An, Vadim Kovalyuk, Alexander Golikov, Yury Gladush, Aram Mkrtchyan, Dmitry Chermoshentsev, Dmitry Krasnikov, Albert Nasibulin, Gregory Goltsman

**Affiliations:** 1 National Research University Higher School of Economics, 101000 Moscow, Russia; 2 Laboratory of Photonic Gas Sensors, University of Science and Technology MISIS, 119049 Moscow, Russia; pashan3000@gmail.com (P.A.);; 3 FRC Institute of Applied Physics RAS, 603950 Nizhny Novgorod, Russia; 4 Department of Physics, Moscow State Pedagogical University, 119992 Moscow, Russia; 5 Skolkovo Institute of Science and Technology, Nobel Street 3, 143026 Moscow, Russiaa.nasibulin@skoltech.ru (A.N.); 6 Russian Quantum Center, 143025 Moscow, Russia

**Keywords:** carbon nanotubes, silicon nitride, photonic integrated circuits, bolometer

## Abstract

The integration of low-dimensional materials with optical waveguides presents promising opportunities for enhancing light manipulation in passive photonic circuits. In this study, we investigate the potential of aerosol-synthesized single-walled carbon nanotube (SWCNT) films for silicon nitride photonic circuits as a basis for developing integrated optics devices. Specifically, by measuring the optical response of SWCNT-covered waveguides, we retrieve the main SWCNT film parameters, such as absorption, nonlinear refractive, and thermo-optic coefficients, and we demonstrate the enhancement of all-optical wavelength conversion and the photoresponse with a 1.2 GHz bandwidth.

## 1. Introduction

Silicon nitride is a highly versatile material that is ideal for integrated photonics due to its wide bandgap of around 5 eV [1], excellent mechanical properties, and compatibility with the CMOS fabrication process [2]. This material is characterized by low infrared light propagation losses, which facilitate the fabrication of passive elements, such as splitters, multiplexers, arrayed waveguide gratings, and filters, all of which exhibit desirable characteristics with low insertion losses [3]. When other materials are incorporated into the integrated circuit, the functionality of the platform can be significantly expanded. Low-dimensional materials can be successfully integrated with photonic waveguides via simple mechanical transfer, and, unlike bulk materials, they do not require lattice matching and do not suffer from interface defects [4]. Successful demonstrations of this approach include ultrafast graphene modulators [5,6,7,8], 2D-material-based photodetectors [9,10,11], carbon nanotube single-photon emitters [12], and photonic van der Waals integration [13]. Another promising candidate for a functional waveguide layer is a thin film of single-walled carbon nanotubes (SWCNTs). This type of film has a sufficient mechanical robustness, demonstrates a high photoresponse [14], and demonstrates nonlinearity 5 orders of magnitude higher than that of silicon nitride [15]. Its electronic properties are defined by the chirality of composing SWCNTs and can be varied from metallic to semiconducting behavior. Unlike 2D materials, the variation in the thickness of an SWCNT film does not change the electronic properties of the material, allowing for the control of the degree of interaction of the evanescent mode of the waveguide with a film [16].

Traditional methods for SWCNT film fabrication require the filtration of the SWCNT from the liquid preceded by the purification and centrifugation of SWCNT dispersion. This procedure usually introduces organic contaminations that reduce the thermal stability.

In our work, we use thin SWCNT films, which are synthesized via floating catalyst (aerosol CVD) synthesis [17]: a one-step method that allows for obtaining pure and uniform organic-free thin films of high-quality carbon nanotubes directly from the reactor. This method also allows for uniform dry-transfer wafer-scale deposition under ambient conditions to be practically applied to any substrate [18].

We transfer the synthesized SWCNT film to a silicon nitride chip on which we fabricate a set of waveguiding structures that allow us to investigate the electrical, optical, and thermo-optical parameters of the films depending on their geometrical parameters. Finally, we demonstrate the applicability of such an approach as a nonlinearity enhancer for a four-wave mixing process and a waveguide-integrated photodetector.

## 2. Materials and Methods

### 2.1. Method for Measuring the Optical and Thermo-Optical Characteristics

**Figure 1:**
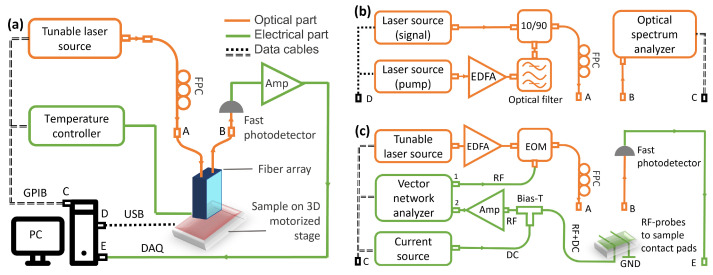
(**a**) Schematic view of the main experimental setup used to characterize fabricated nanophotonic devices. The main setup was used for the optical and thermo-optical characteristics. (**b**) Optical part connected to the main setup to measure the nonlinear optical characteristics. (**c**) Optical and electrical parts connected to the main setup to measure the bolometric photoresponse. A–E—connection points between the main setup and its modifications.

The experimental setup consisted of a tunable laser source (NewFocus TLB-6600), a polarization controller, and an array of optical fibers aligned with the FGCs on the chip using motorized stages (X, Y, Z, and θ) equipped with piezo motors (Figure 1a). To stabilize the temperature of nanophotonic devices, a heater with a thermometer connected to a PID controller (Lakeshore 331) was used. Light from the tunable laser source embedded in an optical fiber, through the polarization controller and the array of optical fibers, entered the input of a nanophotonic device. An array of fibers with the same pitch of 250 μm as for the on-chip input/output FGCs [19] was used. The light passing through the device was detected by a fast photodetector and converted from analog waveforms to digital values (DAQ) [20]. By measuring devices with the same waveguide length but different SWCNT cell widths, the attenuation constants for SWCNT cells of different thicknesses at room temperature were extracted.

In order to measure the thermo-optical coefficient of SWCNT cells on a silicon nitride waveguide, the same procedure as described above was used, but the temperature of the sample was varied in a range of 25–70 ${}^{\circ }$C. By comparing the dependence of the transmission spectrum of an O-ring resonator with and without SWCNT cells, the thermo-optical coefficient was extracted.

### 2.2. Method for Measuring the Nonlinear Characteristics

To measure the nonlinear optical characteristics of the SWCNT cells, we modified the setup shown in Figure 1a by using two narrow-band laser sources (Pure photonics) (Figure 1b). The power of one of the lasers (pump), operated at a wavelength of 1550 nm, was amplified using an erbium-doped optical amplifier (EDFA, KEOPSYS CEFA-C-PB-LP, Lannion, France), filtered, and combined with the second laser source (signal, at 1550.5 nm) through a 90/10 splitter. The light from the chip, including the pump, signal, and idler, was recorded on an optical spectrum analyzer (Yokogawa AQ6370D, 600–1700 nm, Tokyo, Japan).

By using the attenuation constant obtained from the study of the linear characteristics and comparing the powers obtained from the optical spectrum analyzer coming out of the O-ring resonator with and without SWCNT cells of different widths, the nonlinear coefficients were extracted.

### 2.3. Method for Measuring the Bolometric Photoresponse

To measure the bolometric response, we upgraded the experimental setup shown in Figure 1a, replacing the optical and electrical parts as shown in Figure 1c. The optical radiation was amplified using an EDFA and subsequently modulated using a commercial optical modulator (iXBlue MXLN-10, Paris, France). The modulated radiation was then fed into the circuit, where it was absorbed by the SWCNT film acting as a bolometer. The SWCNT cell was biased by the current source (Keithley 2400, Solon, OH, USA) via the bias-T. The bolometric response was amplified and connected to the second channel of a vector network analyzer, VNA (Rohde & Schwarz ZVB 20, Munich, Germany). To sweep the optical modulation, the first port of the VNA was utilized to supply the modulated radiation, covering a frequency range of 0.8 to 7 GHz. Before conducting the measurements, we calibrated the RF part of the setup, including the amplifiers, optical modulator, and coaxes. By using the circuit calibration as a reference and comparing it to the frequency response of the bolometer, the conversion bandwidth of the SWCNT bolometer was extracted.

### 2.4. Fabrication Steps

We fabricated the devices under test using commercially available 540 μm thick silicon wafers, featuring a 2.6 μm thermally oxidized layer topped with a 450 nm layer of Si${}_{3}$N${}_{4}$ deposited via low-pressure chemical vapor deposition (LPCVD).

The first step of our fabrication process involved direct electron lithography (Crestec, CABL -9050C, Tokyo, Japan of MIPT Shared Facilities Center) using a positive electronic resist ZEP520A as a sensitive layer to create optical waveguide devices. We then used the resist developed as a mask during the CHF${}_{3}$ plasma etching of a 225 μm silicon nitride layer. Following this process, we removed any remaining resist using acetone.

**Figure 2:**
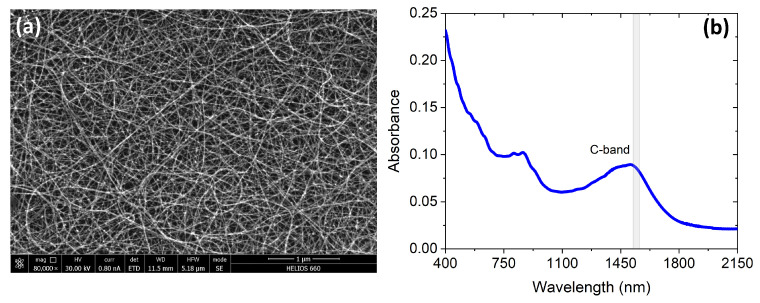
(**a**) SEM image of the SWCNT film used for integration with PICs. (**b**) The dependence of the absorption of the SWCNT film on the wavelength.

The second step involved depositing SWCNT films onto chips synthesized using the aerosol (floating catalyst) chemical vapor deposition method. This process involves the growth of nanotubes on Fe-based aerosol particles through the Boudouard reaction (2CO = C + CO${}_{2}$) within the hot zone of a reactor. The nanotubes are collected on a filter at the outlet of the reactor, forming thin and uniform films that do not require further purification (Figure 2a). SWCNT films can be easily transferred to any surface through the dry-transfer technique [18], with an area of up to A4 paper format [21], allowing for uniform wafer-scale deposition. By placing the film on the filter onto the surface and applying slight pressure, the film transfers to the desired substrate. Not only is this method straightforward, but it is also free from organic contamination, eliminating the need for additional chemical cleaning steps.

In the synthesis, the SWCNT diameter distribution was adjusted via CO/CO${}_{2}$ tuning to ensure that the S${}_{11}$ optical transition maximum occurred at around 1550 nm (Figure 2b).

In the third step, Ti/Au ohmic contacts were formed by using reverse lithography on plates with waveguide devices. Using a positive AZ 1512 as a sensitive layer, a laser lithographer formed the windows of the contact pads. The resist was developed in a 0.7% potassium hydroxide solution. In the developed windows of the resist, an adhesive layer of titanium of 4 nm was evaporated, followed by 100 nm of gold. The lift-off process was performed in acetone.

In the fourth step, rectangular areas of SWCNT films with varying waveguide coverage widths were created. Using a laser lithograph and an AZ 1505 resist, the desired areas of the SWCNT film were exposed. The resist was developed in AZ 726, and the unprotected areas were etched away in oxygen plasma. The remaining resist was removed with acetone, leaving the nanotube films underneath the Ti/Au for good ohmic contact.

**Figure 3:**
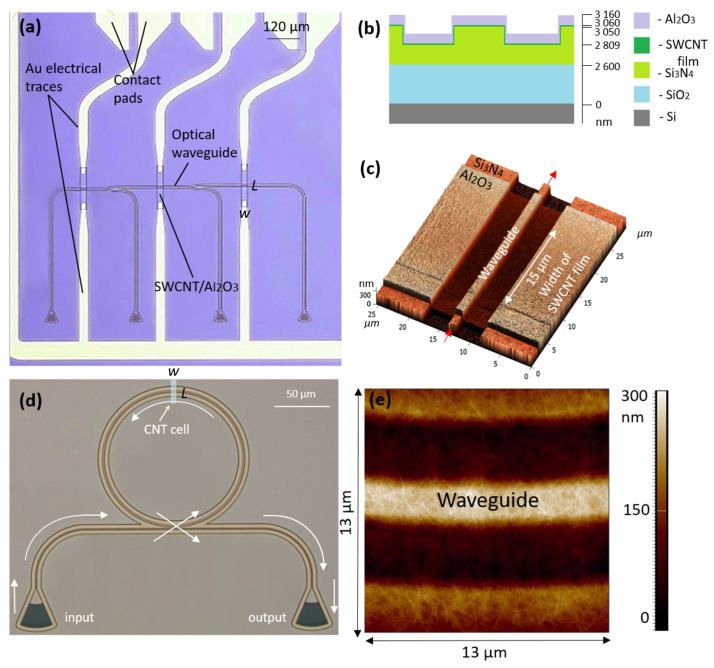
(**a**) Optical micrograph of the fabricated waveguide device with two beam splitters and Au pads. (**b**) Cross-section of the fabricated structure showing layer thicknesses (not shown to scale). (**c**) AFM image of the waveguide, covered by the SWCNT film and Al${}_{2}$O${}_{3}$ film. The arrows schematically show the direction of light. (**d**) Optical micrograph of the O-ring waveguide with the nanotube film on top. The arrows schematically show the direction of light. (**e**) AFM image of the SWCNT film covering part of the O-ring resonator.

To prevent chemical interaction with oxygen, a 100 nm thick layer of aluminum oxide was applied to cover the SWCNT cells in the fifth step. A two-layer MMA/PMMA resist was applied to the plate, and rectangular areas were opened using electronic lithography and a developer diluted with 2-propanol and water in an 8:1 ratio; the opened areas were larger than the SWCNT areas by 10 μm. Al${}_{2}$O${}_{3}$ was deposited by sputtering the electron beam in an Evatec BAK 500 setup, and the lift-off process was carried out with acetone. The cross-sectional illustration in Figure 3b shows the thickness of all layers of the waveguide device, whereas the O-ring resonators did not have oxide layer protection.

## 3. Results

### 3.1. Fabricated Devices

By following the fabrication steps described above, we produced two types of devices.

The first type of device was a series of waveguides featuring two beam splitters coated with thin SWCNT films of the same length (*L* = 100 μm), different cell widths (*w* = 10–65 μm), and a step size of 5 μm (Figure 3a). Each device was connected to gold contacts for electrical measurements and protected with a layer of aluminum oxide on top. Three different sets of such devices, made on different chips and characterized by three different thicknesses ($h_{1}$ = 2 ± 1 nm, $h_{2}$ = 8 ± 4 nm, and $h_{3}$ = 22 ± 4 nm), were used for optical and electrical studies.

To investigate the thermo-optical properties of the SWCNTs, we fabricated the second type of device as an O-ring resonator (Figure 3d). The resulting waveguide featured beam splitters 1 μm in width, with the O-ring resonator waveguide having a width of 1.6 μm, a ring length of 400.4 μm, and a 1.4 μm gap between the ring and the straight waveguide. The widths of the SWCNT film cells atop of the O-rings varied from 100 nm to 650 nm with a step of 50 nm.

In Figure 3c,e, the AFM images show the SWCNT film covering all sides of the rib waveguide, while the nanotubes are intertwined with each other.

### 3.2. Optical Properties of SWCNT Films on a Waveguide

**Figure 4:**
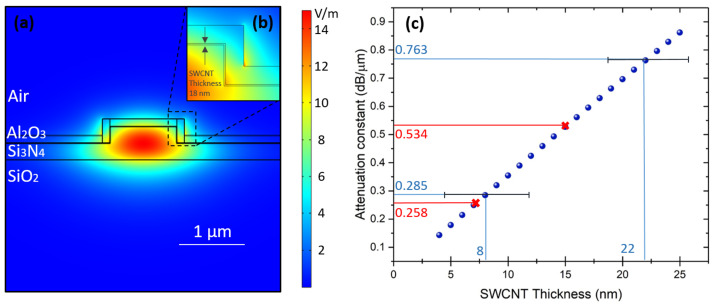
(**a**) The mode profile for the TE-like mode in the Si${}_{3}$N${}_{4}$ waveguide with SWCNT film and Al${}_{2}$O${}_{3}$ film atop. (**b**) Enlarged image of the edge of the waveguide with a nanotube film. (**c**) Simulated change in the attenuation constant of the thickness of the film. Blue marks are simulated values, and red marks are experimental values.

We employed Comsol Multiphysics to calculate the absorption resulting from the evanescent field interaction with the SWCNT film on top of the rib waveguide and investigated various film thicknesses ranging from 4 nm to 25 nm with a step size of 1 nm. The mode distribution with the SWCNT-film-covered silicon nitride waveguide can be observed in Figure 4a,b. Our calculations utilized SWCNTs as a thin film with an effective reflective index at the 1565 nm wavelength (the maximum optical transmission of the fabricated focusing grating couplers (FGCs) [19]). These parameters were obtained from ellipsometry measurements on a similar film [22], yielding *n* = 1.72 and *k* = 0.65. In our model, the nanotube film uniformly covers all sides of the rib waveguide. The calculated results are presented in Figure 4c. As the thickness of the nanotube increases, we observe a linear growth in the attenuation coefficient. In our experimental setup, we used films with thicknesses of 2 ± 1 nm, 8 ± 4 nm, and 22 ± 4 nm. According to the simulations, the expected attenuation coefficients are definitively below 0.1 dB/μm for the thinnest film, and they are approximately 0.29 ± 0.14 dB/μm and 0.76 ± 0.14 dB/μm for the others.

**Figure 5:**
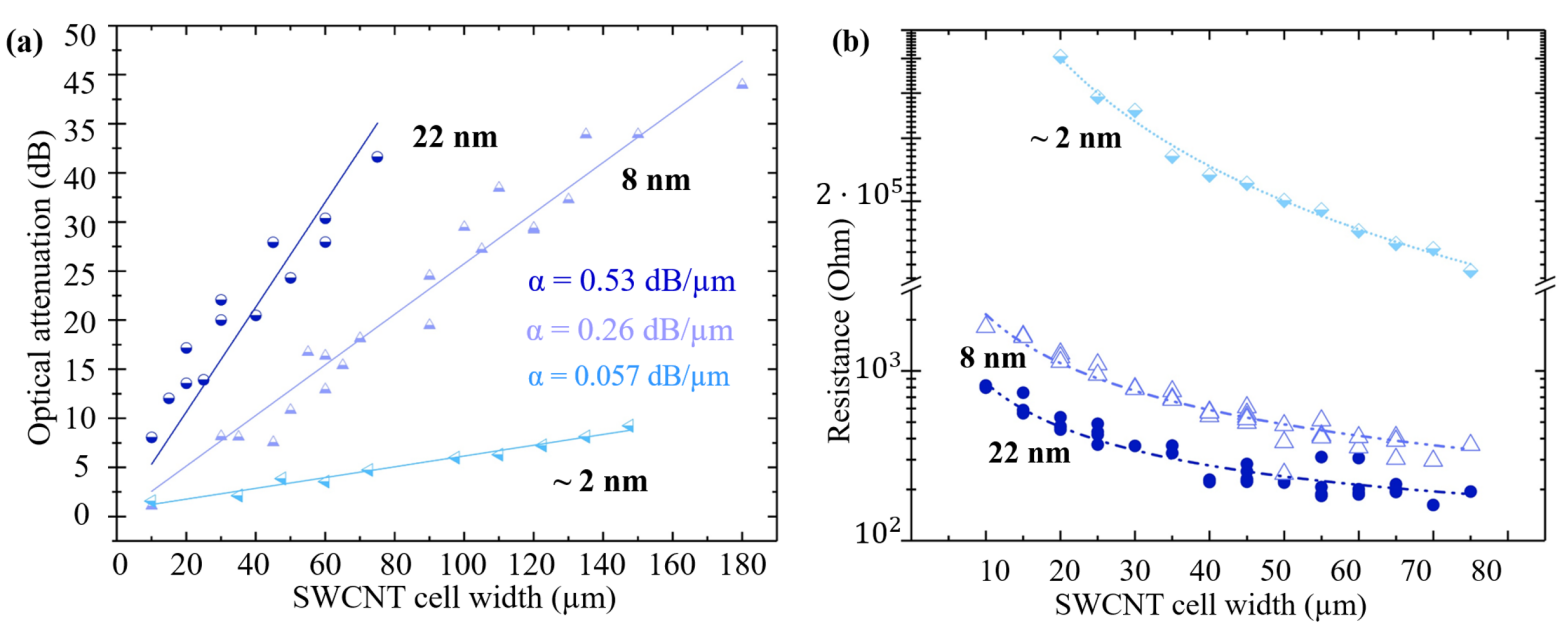
(**a**) Dependence of optical attenuation of light on the width of the SWCNT film for thicknesses $h_{1}$ = 2 ± 1 nm, $h_{2}$ = 8 ± 4 nm, $h_{3}$ = 22 ± 4 nm. Points are experimental data. Lines are linear approximations. (**b**) Width dependence of the resistance of SWCNT films for the same thicknesses. Circles, squares and triangles —experimental data; dashed lines—hyperbolic fit.

To evaluate the absorption of the SWCNT films, we conducted transmission spectroscopy measurements on the waveguides before and after the deposition of the SWCNT films. By comparing the transmittance of the devices before ($T_{b}$) and after ($T_{a}$) the deposition of the SWCNT films at λ = 1565 nm,

(1) $$A\left(w\right)=10\times \log{}_{10}\left(\frac{T_{b}}{T_{a}}\right),$$

we obtained the dependence of attenuation (*A*) on SWCNT cell width. The attenuation for three different thicknesses is shown in Figure 5a. We applied a linear approximation to the dependence of attenuation on the thickness of the film, which yielded attenuation coefficients of $\mu_{1}$ = 0.057±0.002 dB/μm for a 2 nm thick film, $\mu_{2}$ = 0.257±0.005 dB/μm for an 8 nm thick film, and $\mu_{3}$ = 0.534±0.026 dB/μm for a 22 nm thick film.

### 3.3. Electrical Properties of SWCNT Film on a Waveguide

To investigate the impact of lithography on the properties of the SWCNT films, we conducted resistance measurements on films of varying thicknesses as a function of the width of the film. To perform these measurements, we utilized a Keithley 2400 sourcemeter in constant-current mode. Our findings reveal a hyperbolic decrease in resistance as the width of the waveguide coating with films increases:

(2) $$R\left(w\right)=\Omega_{0}+\frac{k^{*}}{w},$$

where $k^{*}$ = ρ·L/h, $\Omega_{0}$—contact resistance, ρ—resistivity, and *L*—SWCNT cell length.

Figure 5b shows the dependence of the resistance of the nanotubes on the width R(w) for the available film thicknesses. The hyperbolic fit gives the values $\Omega_{0}$ = 86 Ohm, $k^{*}$ = 7.6 × 10${}^{-3}$ Ohm·m for $h_{1}$ = 22 nm; $\Omega_{0}$ = 66 Ohm, $k^{*}$ = 21 × 10${}^{-3}$ Ohm·m for $h_{2}$ = 8 nm; and $\Omega_{0}$ = 86 Ohm, $k^{*}$ = 10 Ohm·m for $h_{2}$ = 2 nm. The extracted conductivity values of the films are 60 S·cm${}^{-1}$ for 2 nm, 7180 S·cm${}^{-1}$ for 8 nm, and 7139 S·cm${}^{-1}$ for 22 nm, showing good agreement between each other. The drop in conductivity for the 2 nm film can be attributed to a reduced percolation network.

### 3.4. Nonlinear Optical Properties of SWCNT Film on a Waveguide

**Figure 6:**
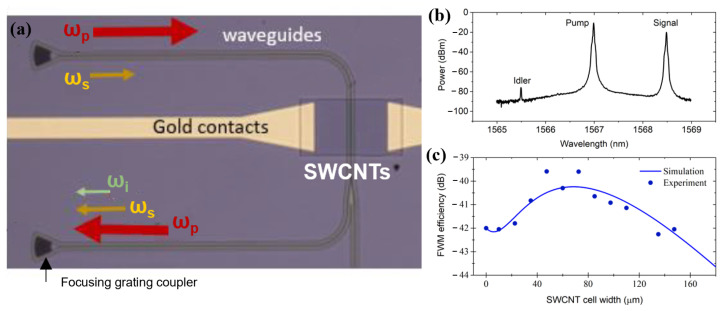
(**a**) Illustration of idler generation through the waveguide with SWCNT film atop. (**b**) The DFWM spectrum (pump, signal, idler) obtained from an optical spectrum analyzer. (**c**) Dependence of the four-wave mixing efficiency on the width of the SWCNT cell width ($h_{1}$ = 2 ± 1 nm). The dot curve represents the experimental data, and the solid line represents the simulation, using Equation (4).

Using the experimental setup shown in Figure 1b, we investigated the enhancement of degenerate four-wave mixing (DFWM) with an SWCNT film on the waveguide, which is defined by third-order susceptibility. In DFWM (frequency domain), the interaction of the pump ($\omega_{p}$) and signal ($\omega_{s}$) gives rise to an idler ($\omega_{i}$) wave, defined by the relation $\omega_{i}=2\cdot \omega_{p}-\omega_{s}$. (Figure 6a). The efficiency of the wavelength conversion η was defined as follows:

(3) $$\eta =10\times \log{}_{10}\left(\frac{P_{i}\left(z=w\right)}{P_{s}\left(z=0\right)}\right),$$

where $P_{i}$ and $P_{s}$ are the idler and signal powers, respectively, and *z* is the distance along the waveguide. The SWCNT film increases the efficiency of the nonlinear optical conversion but, at the same time, introduces additional losses to the system. This leads to a strong nonmonotonic dependence of the conversion efficiency on the SWCNT film width. To obtain the nonlinear coefficient of the SWCNTs, we compared our experimental results with the numerical solution of a set of equations that defines the evolution of the pump, signal, and idler waves in the SWCNT-covered waveguide:

$$\frac{dE_{p}}{dz}=i\gamma (|E_{p}{|}^{2}+2|E_{s}{|}^{2}+2|E_{i}{|}^{2})E_{p}+2i\gamma E_{p}^{*}E_{s}E_{i}e^{i\Delta \beta z}-\frac{1}{2}\alpha E_{p},$$

(4) $$\frac{dE_{s}}{dz}=i\gamma (|E_{s}{|}^{2}+2|E_{i}{|}^{2}+2|E_{p}{|}^{2})E_{s}+i\gamma E_{i}^{*}E_{p}^{2}e^{-i\Delta \beta z}-\frac{1}{2}\alpha E_{s},$$

$$\frac{dE_{i}}{dz}=i\gamma (|E_{i}{|}^{2}+2|E_{s}{|}^{2}+2|E_{p}{|}^{2})E_{i}+i\gamma E_{s}^{*}E_{p}^{2}e^{-i\Delta \beta z}-\frac{1}{2}\alpha E_{i},$$

where $E_{x}$ are the electric fields of the waves, α is the attenuation constant measured in the previous section, $\Delta \beta =\beta_{2}\Delta \omega^{2}$ is the phase mismatch, and $\beta_{2}$ is the waveguide second-order dispersion coefficient. On the right-hand side, the first three terms are responsible for self-phase and cross-phase modulations, the fourth term is for the FWM, and the last term is for the losses. The nonlinearity parameter γ of the SWCNT film on the waveguide was chosen by fitting the simulation curve to the experiment.

We found that only waveguides covered with the thinnest SWCNT film (2 nm) provide the enhancement of conversion efficiency, while the thicker films provide too strong an absorption. To determine the optimal length of the SWCNT film, the length of the covered SWCNT film was varied in the range from 10 to 180 μm.

Figure 6b represents the experimental efficiency (dot curve) and simulated efficiency (solid line) of FWM depending on the length of the SWCNT at the 45 mW pump power in the waveguide. The efficiency −42 dB at the zero SWCNT film length corresponds to the uncovered waveguide. We found that the waveguide covering of the film with lengths of up to 120 μm leads to an improvement in the conversion efficiency, reaching its maximum at 70 μm with a greater enhancement of more than 2 dB. In the simulation, we also took into account small idler generation on the fibers preceding the Si${}_{3}$N${}_{4}$ waveguide and estimated the nonlinearity parameter $\gamma =9800\pm 2400\;W^{-1}m^{-1}$, which is 3 orders of magnitude higher than the nonlinearity of the bare silicon nitride waveguide. Then, using Comsol Multiphysics, we calculated the nonlinear refractive index of the nanotubes. Parameter γ depends on the overlap of the mode field with the carbon nanotube cover and is defined by the following integral:

(5) $$\gamma =\frac{2\pi }{\lambda }\frac{{\;\int \int \;}_{D}\;n_{0}^{2}\left(x,y\right)n_{2}\left(x,y\right)E^{4}\left(x,y\right)dxdy}{{\left({\;\int \int \;}_{D}\;n_{0}\left(x,y\right)E^{2}\left(x,y\right)dxdy\right)}^{2}},$$

where *D* is the integration over the cross-section of the waveguide and SWCNT film, and $n_{2}$ is different for Si${}_{3}$N${}_{4}$ and SWCNTs. Taking the nonlinear coefficient of Si${}_{3}$N${}_{4}$ as being equal to $10^{-18}$ m${}^{2}$W${}^{-1}$, we find the nonlinear coefficient of the SWCNT film $n_{2}$(SWCNT) $=\left(1.5\pm 0.4\right)\times 10^{-13}$ m${}^{2}$W${}^{-1}$. This value is in good agreement with that in other work on the nonlinear optical response of SWCNTs [15].

### 3.5. Thermo-Optical Properties of SWCNT Film on a Waveguide

**Figure 7:**
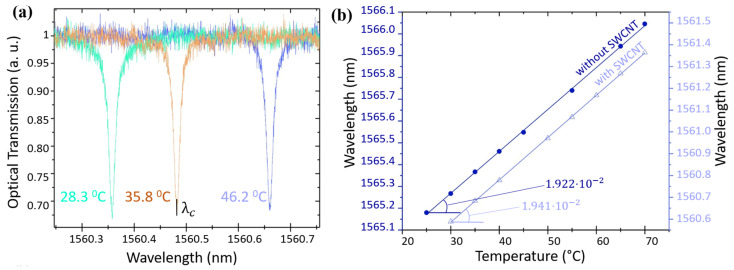
(**a**) Parts of the transmission spectrum of an O-ring resonator with nanotubes with increasing temperature. Each color has a different temperature. (**b**) Change in the resonance wavelength of an O-ring resonator with a change in temperature for a device with nanotubes (triangles) and without nanotubes (circles). The lines are linear fit for each case.

Next, we proceeded to measure the thermo-optical coefficient $\beta^{*}$$\equiv dn_{eff}/dT$ of the SWCNTs by observing the shift in the resonances of ring cavities covered with and without SWCNTs when subjected to chip heating (Figure 7a). Compared to the O-ring resonators without SWCNTs, the shift of the resonant wavelength occurs faster (Figure 7b), indicating that this system is sensitive enough for measuring the thermo-optical coefficient of the SWCNT film.

First, by using the values of the order of interference *m*, the radius of the ring (*R*), and the shift of the resonant wavelength ($\lambda_{c}$), we extracted the effective refractive index of the entire structure by using the following formula:

(6) $$\Delta n_{eff}\left(T\right)=\frac{m\Delta \lambda (T)}{2\pi R}.$$

The value $\beta^{*}$ extracted from the linear fit was 1.66 ×$10^{-5}$ RIU/${}^{\circ }$C. Then, using the literature data [23] for $\beta^{*}$(Si${}_{3}$N${}_{4}$) = 2.51 ×$10^{-5}$, $\beta^{*}$(SiO${}_{2}$) = 0.96 × 10${}^{-6}$ based on the numerical simulation in COMSOL Multiphysics, we found values $\beta^{*}$(SWCNT) that agree with the experimental data on the change in the effective refractive index. The values obtained for the temperature range from 25 ${}^{\circ }$C to 70 ${}^{\circ }$C is $\beta^{*}$(SWCNT) = 2.02 × 10${}^{-6}$ RIU/${}^{\circ }$C.

### 3.6. Photoresponse of SWCNT Film on a Waveguide

Finally, we investigated the capability of the SWCNT films to detect optical radiation in the bolometric regime. To achieve this, we made several modifications to our experimental setup by adding an RF circuit (Figure 1c).

**Figure 8:**
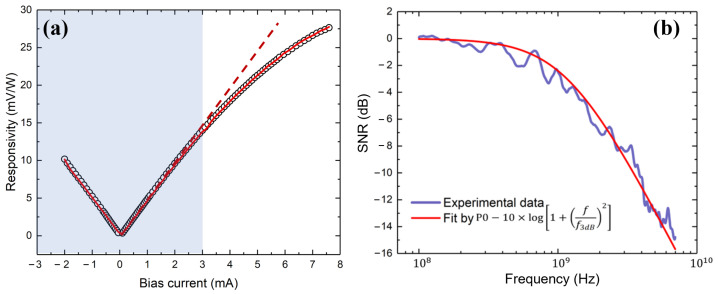
(**a**) Dependence of responsivity for device with *w* = 30 μm width vs. biasing current (black circles). The region highlighted in color corresponds to a linear dependence on the bias current. The red dotted line is a linear fit. (**b**) Normalized responsivity of the structure with 30 μm width (purple line) and fitting (red line) by which the cutoff frequency was determined and equal for this bolometer at 1.2 GHz.

By applying an electrical signal (100 kHz) from a vector network analyzer to an electro-optical modulator, we first measured the dependence of the bolometer responsivity on the bias current (Figure 8a). The obtained dependence shows linear and nonlinear regions. The linear region corresponds to quasi-equilibrium measurement conditions (blue region and dashed red line as a line fit), and the nonlinear region, where the responsivity saturates, is associated with the phenomenon of self-heating and an increase in the resistance of the bolometer. By selecting a bias current of $I_{b}$ = 2 mA within the linear region, we tuned electrical modulation in the range from 10 kHz to 20 GHz, and we directly measured the bandwidth of the bolometer. Figure 8b shows the typical normalized responsivity R of a bolometer with *w* = 30 μm, *L* = 100 μm, and an SWCNT film thickness of 8 nm. The purple line shows the measured data, and red one is the fitting with a function of the following form:

(7) $$P=P_{0}-10\times \log{}_{10}\left(1+{\left(\frac{f}{f_{3\mathrm{dB}}}\right)}^{2}\right),$$

where $P_{0}$ is the value of the bolometer response on the plateau (low frequencies), and $f_{3\mathrm{dB}}$ is the fitting parameter, which allows for the cutoff frequency to be determined as the point where $P_{0}$ is reduced by 3 dB. On the basis of the results of the described fitting, $f_{3\mathrm{dB}}$ = 1.2 GHz was obtained for the considered integrated bolometer.

Furthermore, we determined another crucial parameter of bolometers, known as the noise equivalent power (NEP). NEP is defined as the ratio of noise spectral density $S_{n}$ to responsivity R: $\mathrm{NEP}=S_{n}/R$. Essentially, NEP represents the optical power level at which the generated signal becomes equal to the intrinsic noise of the bolometer. In our case, the parameter $S_{n}$ was determined with the laser source switched off; therefore, it was found that, for the bolometer, the parameter NEP = 5.1 $\times \;10^{-7}$ W${\mathrm{Hz}}^{-1/2}$.

Finally, we defined another important parameter that is used to characterize bolometers, namely, specific detectivity $D^{*}$, as the ratio of the root of the bolometer area *S* to NEP: $D^{*}=\sqrt{S}/\mathrm{NEP}$. We found that, for the studied bolometer, $D^{*}=1.1\times 10^{4}$ cmHz${}^{1/2}$ W${}^{-1}$.

## 4. Discussion

Table 1 summarizes the results of the measurements of the main parameters of the SWCNT films, which are necessary for designing and complicating hybrid photonic-SWCNT devices in the future. While the attenuation coefficient (μ) for the thin film agrees well with that in the simulation, the value of the thicker film falls outside of the expected range. This discrepancy may be attributed to uncertainties in the film thickness and refractive index. In contrast, thicker films are well suited to the dependence of resistance on thickness, whereas a 2 nm thick film has a higher resistance. The drop in conductivity for the 2 nm film can be attributed to a reduced percolation network.

Carbon nanotubes are known to demonstrate a high nonlinear optical response, which is several orders of magnitude higher than that of standard waveguiding materials, such as silicon oxide, silicon nitride, and silicon [15]. Integrating an SWCNT film in a waveguide can significantly increase the nonlinearity of the system [24]. Here, we investigated the enhancement of degenerated four-wave mixing (DFWM) with an SWCNT film on a waveguide, which is defined by third-order susceptibility. The FWM phenomenon is used for various applications, such as all-optical wavelength conversion for division multiplexing in telecommunication and the generation of frequency combs in integrated O-ring resonators. By taking the nonlinear refractive index of Si${}_{3}$N${}_{4}$ as being equal to $10^{-18}$ m${}^{2}$W${}^{-1}$, we found the nonlinear refractive index of carbon nanotubes $\left(1.5\pm 0.4\right)\times 10^{-13}$ m${}^{2}$W${}^{-1}$. This value is in good agreement with that in other work on the nonlinear optical response of SWCNTs [15]. It is also slightly higher than that obtained for graphene-covered SiN waveguides with similar losses of 0.05 dB/μm [25,26]. Here, we used an effective SWCNT film thickness of 2 nm, which provides a higher nonlinear coefficient simply due to the large thickness compared to single-layer graphene. A further increase in the SWCNT thickness can provide an even larger nonlinear coefficient but also larger losses, decreasing the final conversion efficiency.

The considered bolometer showed a response time that was six orders of magnitude faster than that of the free-standing film of similar SWCNTs, at the cost of slightly lower detectivity [27]. Here, we especially note that, in this study, parameters such as R, NEP, and $D^{*}$ were significantly influenced by the impedance mismatch between the bolometer resistance and the equipment’s standard 50 Ohm wave impedance. Consequently, by modifying the geometry of the bolometers, it is possible to achieve significant increases in these parameters.

## 5. Conclusions

Our results demonstrate that aerosol-synthesized SWCNT thin films allow for convenient and robust implementation on waveguides, supporting wafer-scale deposition under ambient conditions and withstanding the necessary lithography steps without compromising their properties.

We measured various characteristics of hybrid nanophotonic circuits covered by SWCNT films, including conductivity, the thermo-optic coefficient, and the nonlinear optical coefficient. In particular, the SWCNT film exhibited a significant enhancement in nonlinearity, with a three-fold increase in nonlinear parameters compared to the bare waveguide, reaching $\gamma =9800\pm 2400\;W^{-1}m^{-1}$. The introduced losses varied from 0.057 dB/m for a 2 nm film thickness to 0.53 dB/m for 22 nm. The losses represent the limiting factor for the wavelength conversion efficiency, which could be increased on more than 2 dB with a 70 μm long 2 nm thick film in a four-wave mixing experiment.

The high conductivity and pronounced thermal coefficient of the SWCNT film make it suitable for application as a bolometric photodetector. We achieved a noise equivalent power NEP = 5.1 × 10${}^{-7}$ W/$\sqrt{\mathrm{Hz}}$ and a specific detectivity $D^{*}=1.1\times 10^{4}$ cmHz${}^{1/2}$ W${}^{-1}$, with a cutoff frequency of 1.2 GHz. When the parameters and geometry of the SWCNT film are further fine-tuned, we can significantly enhance the performance of hybrid photonic devices covered with SWCNT films.

## Figures and Tables

**Table 1:** Optical and electrical parameters of hybrid PICs covered by SWCNT films.

Film Thickness, nm	$k^{*}$, Ohm·m	μ, dB/μm	γ, W${}^{-1}$m${}^{-1}$	$D^{*}$, cmHz${}^{1/2}$W${}^{-1}$
2 ± 1	10	0.057±0.002	9800 ± 2400	-
8 ± 4	20 × 10${}^{-3}$	0.257±0.005	-	$1.1\times 10^{4}$
22 ± 4	7.6 × 10${}^{-3}$	0.534±0.026	-	-

## Data Availability

The data presented in this study are available on request from the corresponding author.

## Abbreviations

The following abbreviations are used in this manuscript:

AFM	Atomic force microscope
CMOS	Complementary metal oxide semiconductor
DFWM	Degenerated four-wave mixing
EDFA	Erbium-doped fiber amplifier
FGC	Focusing grating coupler
FWM	Four-wave mixing
LPCVD	Low-pressure chemical vapor deposition
MIPT	Moscow Institute of Physics and Technology
NEP	Noise equivalent power
PIC	Photonic integrated circuit
SWCNT	Single-walled carbon nanotube
VNA	Vector network analyzer

## References

[1] Blumenthal D.J., Heideman R., Geuzebroek D., Leinse A., Roeloffzen C. (2018). Silicon Nitride in Silicon Photonics. Proc. IEEE.

[2] Muñoz P., Micó G., Bru L.A., Pastor D., Pérez D., Doménech J.D., Fernández J., Baños R., Gargallo B., Alemany R. (2017). Silicon Nitride Photonic Integration Platforms for Visible, Near-Infrared and Mid-Infrared Applications. Sensors.

[3] Xiang C., Jin W., Bowers J.E. (2022). Silicon nitride passive and active photonic integrated circuits: Trends and prospects. Photonics Res..

[4] Wu J., Ma H., Yin P., Ge Y., Zhang Y., Lil L., Zhang H., Lin H. (2021). Two-dimensional materials for integrated photonics: Recent advances and future challenges. Small Sci..

[5] Liu M., Yin X., Ulin-Avila E., Geng B., Zentgraf T., Ju L., Wang F., Zhang X. (2011). A graphene-based broadband optical modulator. Nature.

[6] Dalir H., Xia Y., Wang Y., Zhang X. (2016). Athermal broadband graphene optical modulator with 35 GHz speed. ACS Photonics.

[7] Phare C., Lee Y., Cardenas J., Lipson M. (2015). Graphene electro-optic modulator with 30 GHz bandwidth. Nat. Photonics.

[8] Sun Z., Martinez A., Wang F. (2016). Optical modulators with 2D layered materials. Nat. Photonics.

[9] Schuler S., Schall D., Neumaier D., Dobusch L., Bethge O., Schwarz B., Krall M., Mueller T. (2016). Controlled Generation of a p–n Junction in a Waveguide Integrated Graphene Photodetector. Nano Lett..

[10] Youngblood N., Chen C., Koester S., Li M. (2015). Waveguide-integrated black phosphorus photodetector with high responsivity and low dark current. Nat. Photonics.

[11] Bie Y., Grosso G., Heuck M., Furchi M., Cao Y., Zheng J., Bunandar D., Navarro-Moratalla E. (2017). MoTe2-based light-emitting diode and photodetector for silicon photonic integrated circuits. Nat. Nanotechnol..

[12] Khasminskaya S., Pyatkov F., Słowik K., Ferrari S., Kahl O., Kovalyuk V., Rath P., Vetter A., Hennrich F., Kappes M. (2016). Fully integrated quantum photonic circuit with an electrically driven light source. Nat. Photonics.

[13] Meng Y., Feng J., Han S., Xu Z., Mao W., Zhang T., Kim J.S., Roh I., Zhao Y., Kim D.H. (2023). Photonic van der Waals integration from 2D materials to 3D nanomembranes. Nat. Rev. Mater..

[14] Itkis M., Borondics F., Yu A., Haddon R. (2006). Bolometric Infrared Photoresponse of Suspended Single-Walled Carbon Nanotube Films. Science.

[15] Yamashita S. (2019). Nonlinear optics in carbon nanotube, graphene, and related 2D materials. APL Photonics.

[16] Davletkhanov A.I., Mkrtchyan A.A., Chermoshentsev D.A., Shashkov M.V., Ilatovskii D.A., Krasnikov D.V., Nasibulin A.G., Gladush Y.G. (2023). Inverted loss engineering in functional material covered waveguides. arXiv.

[17] Khabushev E.M., Krasnikov D.V., Zaremba O.T., Tsapenko A.P., Goldt A.E., Nasibulin A.G. (2019). Machine learning for tailoring optoelectronic properties of single-walled carbon nanotube films. J. Phys. Chem. Lett..

[18] Kaskela A., Nasibulin A.G., Timmermans M.Y., Aitchison B., Papadimitratos A., Tian Y., Zhu Z., Jiang H., Brown D.P., Zakhidov A. (2010). Aerosol-synthesized SWCNT networks with tunable conductivity and transparency by a dry transfer technique. Nano Lett..

[19] Kuzin A., Kovalyuk V., Golikov A., Prokhodtsov A., Marakhin A., Ferrari S., Pernice W., Gippius N., Goltsman G. Efficiency of focusing grating couplers versus taper length and angle. Proceedings of the 6th International School and Conference “Saint Petersburg OPEN 2019”: Optoelectronics, Photonics, Engineering and Nanostructures.

[20] Komrakova S., Kovalyuk V., An P., Golikov A., Rybin M., Obraztsova E., Goltsman G. (2020). Effective absorption coefficient of a graphene atop of silicon nitride nanophotonic circuit. J. Phys. Conf. Ser..

[21] Gubarev V., Yakovlev V., Sertsu M., Yakushev O., Krivtsun V., Gladush Y.G., Ostanin I., Sokolov A., Schäfers F., Medvedev V. (2019). Single-walled carbon nanotube membranes for optical applications in the extreme ultraviolet range. Carbon.

[22] Ermolaev G.A., Tsapenko A.P., Volkov V.S., Anisimov A.S., Gladush Y.G., Nasibulin A.G. (2020). Express determination of thickness and dielectric function of single-walled carbon nanotube films. Appl. Phys. Lett..

[23] Elshaari A.W., Zadeh I.E., Jöns K.D., Zwiller V. (2016). Thermo-Optic Characterization of Silicon Nitride Resonators for Cryogenic Photonic Circuits. IEEE Photonics J..

[24] Chow K., Yamashita S. (2010). Four-wave-mixing-based wavelength conversion using a single-walled carbon-nanotube-deposited planar lightwave circuit waveguide. Opt. Lett..

[25] Alexander K., Savostianova N.S., Mikhalov B.K., Thourhout D. (2017). Electrically tunable optical nonlinearities in graphene covereds in waveguides characterized by four-wave mixing. ACS Photonics.

[26] Vermeulen N. (2022). Perspectives on nonlinear optics of graphene: Opportunities and challenges. APL Photonics.

[27] Kurtukova T.N., Kopylova D.S., Raginov N.I., Khabushev E.M., Novikov I.V., Serebrennikova S.I., Krasnikov D.V., Nasibulin A.G. (2023). Plasma-treated carbon nanotubes for fast infrared bolometers. Appl. Phys. Lett..

